# A new method to predict the outcome of the volar locked plate treatment for distal radius fracture

**DOI:** 10.1186/s12891-019-2931-3

**Published:** 2019-11-13

**Authors:** Hui Yao, Weijia Zhang, Wenbin Xu, Kaihua Liu, Yichun Xu

**Affiliations:** 1 Department of Orthopaedics, The Third Affiliated Hospital of Sun Yat-sen University, NO. 2693 Kaichuang Road, Guangzhou, 510100 China; 2 School of Pharmacy, Xinhua College of Sun Yat-sen University, Guangzhou, China

**Keywords:** Wrist fracture, Volar plating, Outcome evaluation, Prediction method

## Abstract

**Background:**

Volar locked plate for distal radius fracture is one of the common procedures performed in trauma surgery. There are already some factors which can be used to predict the functional outcome after volar locked plating for distal radius fracture. However their limitations caused that the outcomes couldn’t be satisfactorily predicted. Better factors for predicting the prognosis more precisely are of great interest. The aim of this study is to introduce such a new factor.

**Methods:**

A total of 56 patients suffered from unilateral distal radius fracture were managed operatively with the volar locked plate. Before operation, all CT scans of the distal radius were obtained. The ratios of soft tissue circumference to bone circumference at the watershed line in the distal radius were calculated based on the preoperative CT scans. Outcomes were evaluated after operation. The correlations between the ratio and the outcomes were analyzed using single factor linear regression analysis.

**Results:**

Statistically significant linear relationships between the ratio and flexion degrees, extension degrees also patient-rated wrist evaluation (PRWE) scores were discovered. With the increase of the ratios, the flexion and extension range increased and the PRWE scores declined.

**Conclusions:**

There are obvious linear relationships between the ratio and postoperative wrist flexion-extension degrees also PRWE scores when using volar locked plating for distal radius fracture. So the ratio can be used as a predictor aiding surgeons to predict the outcome.

## Background

The distal radius fracture (DRF) is very common since it accounts for 44% of all types of hand and forearm fractures [1] and 20% of all fractures [2].

Volar locked plate (VLP) fixation for DRF has increasingly gained popularity since the use of fixed-angle screws within volar plates provides satisfactory stability [3, 4] and allows early wrist mobilization especially for the comminuted intra-articular fracture or the osteoporosis bone. Patients recover quicker after volar locked plating than other managements [5, 6]. So the volar locked plating technique has been widely applicated and many encouraging functional outcomes have been reported [7, 8].

Nevertheless, the volar locked plating technique has its own drawbacks, such as large soft tissue dissection, tendinous damage, median nerve injury, hardware complications and pain [9], which compromise the clinical results directly or indirectly. So how to precisely predict the outcomes of VLP treatment for DRF remains a problem.

There are already some factors reported to predict the functional outcomes following DRF. Myderrizi N [10] found that factors such as age, AO classification, distal radial ulnar joint injury, ulnar styloid fracture and initial displacement are predictive of reduction loss. Belloti JC et al. [11] also found that ulnar styloid fracture may be a predictive factor of worse functional outcome for DRF. Cheecharern S [12] pointed out that age, sex and size of dorsal cortex comminution can be used to predict the late dorsal tilt angulation of distal articular surface of radius at the end of the immobilization. Lutz M et al. [13] advocated an increase in articular cavity depth and anteroposterior distance of the lunate fossa should be avoided when performing plate fixation to improve results following distal intra-articular radius fractures.

That is to say, all of these factors such as age, sex, type of fracture, surgical manipulation and reduction quality can be used to predict the outcomes following DRFs. But when using these factors for prediction, it depends mainly on surgeons’ experience and there aren’t many specific standards. Now we introduce a new index which can be measured in figures and calculated precisely to predict the outcomes of VLP treatment for DRF. That is the ratio of soft tissue circumference to bone circumference (STCTBC) at the watershed line in the distal radius. The aim of this study is to report our research findings about this method.

## Methods

A total of 56 patients suffered from unilateral DRFs were managed by a single well trained surgeon using open reduction and internal fixation with a VLP from October 2011 to March 2015. There were 26 males and 30 females with the mean age 54 (from 34 to 77). All patients gave consent to take part in the research and the study was approved by the local ethics committee.

Fractures were caused either by high-energy injury including traffic accident in 16 cases and high fall injury in 5, or by low-energy injury including 32 cases of fall in walk and 3 falls in ball games. According to AO classification, there were two A3, 11 B2, nine B3, 10 C1, 21 C2, and three C3. Patients included in this study met the following criteria: extra-articular fractures (A3 Type) with the presence of radial shortening > 3 mm and dorsal tilt> 10 degrees; intra-articular fractures (Type B2, B3, C1, C2, C3) with step-off > 2 mm; all fractures were fixed with VLP. Open fractures and fractures treated conservatively, accompanied with tendon damage or nerve injury immediately after trauma, fixed with other implants such as external fixator, K-wires, bone grafts were excluded.

**Fig. 1:**
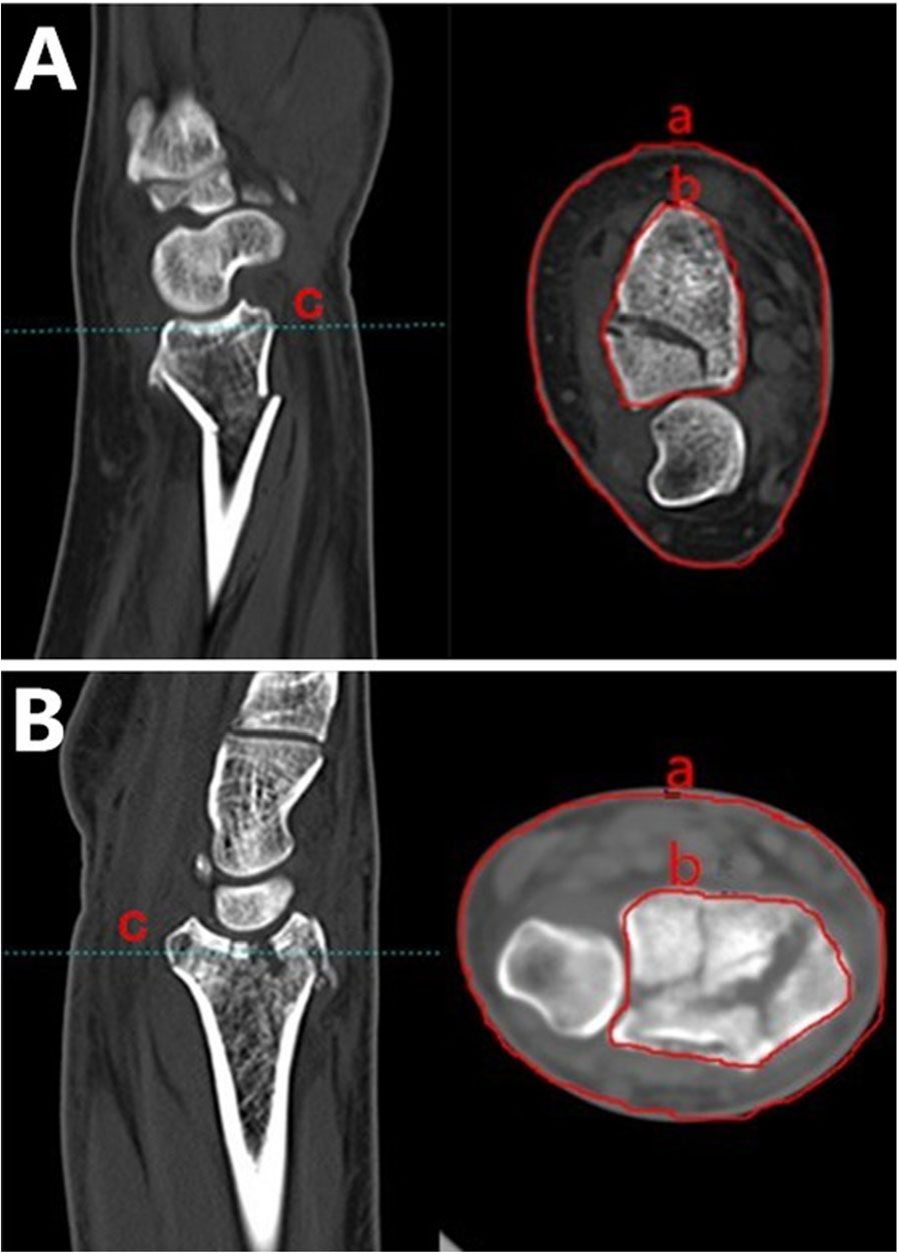
**A** type C1, **B** Type B2. a: soft tissue circumference at the watershed line; b: bone circumference at the watershed line; c: locating line through the most prominent point in the sagittal plane; The ratio = a/b

On the day of admittance, patients’ demographic and clinical data were recorded and physical examination and initial radiographic evaluation were performed. All fractures were reset and temporarily maintained with casts by a single well trained chief resident. The ratios of STCTBC at the watershed line were calculated based on the preoperative CT scans taken 5 to 7 days after injury (just before the surgery) with the cast removed when the posttraumatic edema had completely subsided. The measurements were performed in the imaging system of our hospital (Yilianzhong PACS System, Version 2014.12.03.0). Firstly, the cross section for circling the edge of bone and soft tissue should be found. Among all the sagittal planes, find the one which includes the most prominent point in the volar side. The cross section corresponding to the locating line (line “c” in the Fig. 1) which goes through the most prominent point in the sagittal plane was just the transverse section wanted for circling the edge of bone and soft tissue. Secondly, in the cross section located, a curve tightly around the edge of the skin surface (line “a” in Fig. 1) was drawn with the help of the system and the length of the curve was provided by the system immediately after completing the curve. In the same way, the curve tightly around the edge of the bone surface of radius (line “b” in Fig. 1) was drawn and its length was obtained. Finally, the length of line “a” divided by the length of line “b” was just the ratio of STCTBC at the watershed line in the distal radius (Fig. 1). The length of line “a” signifies the peau circumference at the watershed line while the length of line “b” indicates the bone circumference at the watershed line in the distal radius. So the ratio of a/b (STCTBC) can be used to indicate the surrounding space between the skin surface and the bone surface at the watershed line in the distal radius. The greater the ratio is, the larger the space is between the skin and bone surface.

Surgical interventions were all performed 5 to 7 days after the time of injury when the posttraumatic edema had completely subsided. The volar approach was applied with flexor carpi radialis and median nerve pulled in the ulnar side and radial artery in the radial side. The soft tissue was carefully protected during operation. After pronator quadratus dissected longitudinally, the fracture was well exposed. With regard to the extra-articular fracture (Type A3), the temporary fixation k-wire was used immediately after the radius length, palmar tilt and radial inclination restored. For the intra-articular fractures (TypeB2, B3, C1, C2, C3), the arthroscopy technique was performed to check the articular surface anatomically restored and confirm the disappearance of the step-off. Locking screws were used in all the distal fracture fragments to support the articular surface. The position of the plate and correct reduction were confirmed using radiographic views. Range of motion and fracture stability were examined before skin closure. Active wrist mobilization began supervised by a physiotherapist immediately after operation.

Radiological follow-up was conducted at 1, 3, 6 months and 1 year after operation. All fractures were followed up until union. At each visit, anteroposterior and lateral radiographs were assessed by an independent radiologist. Radiographic parameters including volar tilt, radial inclination, radial height and ulnar variance were measured at the radiographs taken at the follow-up of 6 months after operation. Objective function was assessed with grip strength, range of motion (flexion, extension, pronation, supination, radial deviation and ulnar deviation) by an independent physiotherapist at 1 year after operation. Grip strength was measured with a dynamometer. Patients were asked to grip the dynamometer as much force as possible using the fractured hand and the maximum reading showed in the dynamometer was just the grip strength. The range of motion was measured using a standard goniometer while patients moved their wrists to the maximum range. Score evaluations including the disabilities of the arm, shoulder and hand (DASH) [14] and the patient-rated wrist evaluation (PRWE) [15] outcome questionnaire were also involved to assess the functional outcome by the physiotherapist mentioned above. Complications were all noted immediately after surgical intervention and then during the whole visits.

SPSS version 16.0 was used to complete the data analysis. Single factor linear regression analysis was performed to analyze the correlation between the ratio of STCTBC at the watershed line in the distal radius and the clinical outcomes. Continuous variables were defined by the mean ± standard deviations. A *p*-value of less than 0.05 was considered to be statistically significant.

## Results

**Fig. 2:**
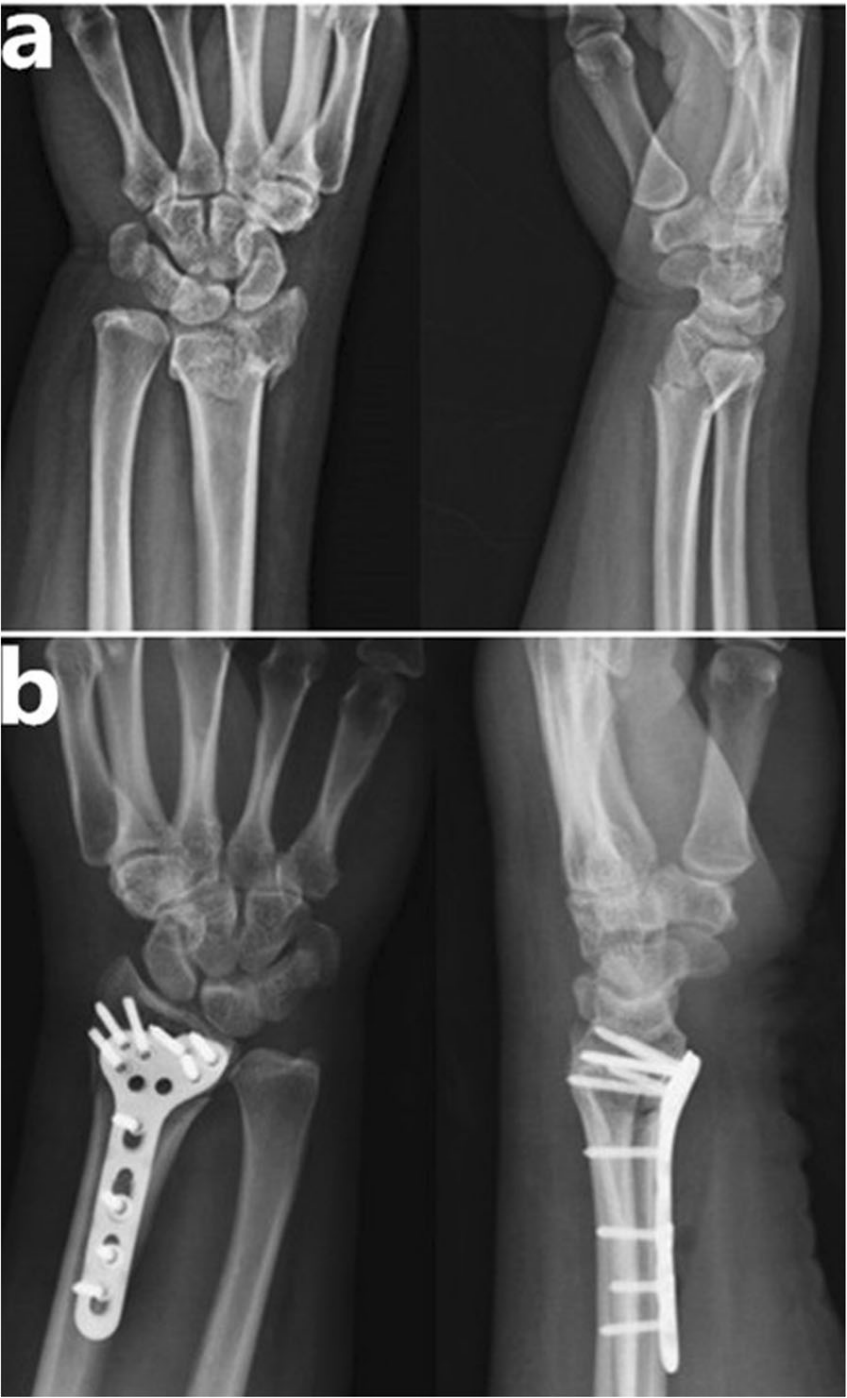
**a** Anteroposterior and lateral radiographs of a 34-year-old female with AO C1 fracture before operation. **b** Posteroanterior and lateral radiographs taken immediately after operation showed that anatomic reduction had been obtained using a VLP

All patients were followed up at least 1 year with no one lost. All fractures united at an average of 13 weeks (range, 10–19 weeks), as determined by clinical examination and follow-up radiographs (Fig. 2).

The ratio of STCTBC at the watershed line in the distal radius measured in the preoperative CT scans was 1.82 ± 0.16 (1.56~2.14).

At the follow-up of the sixth month, the radiographic parameters were as follows: volar tilt (9.45 ± 2.18)°, radial inclination (24.61 ± 3.86)°, radial height (10.73 ± 4.01) mm and ulnar variance (0.4 ± 1.2) mm.

At the follow-up of the twelfth month, the degrees of wrist motion were as follows: flexion 64.63 ± 7.46 (46~77); extension 61.91 ± 7.44 (43~75); radial deviation 17.30 ± 3.52 (10~24); ulnar deviation 21.79 ± 4.01 (13~30); pronation 74.36 ± 8.91 (60~92) and supination 77.43 ± 8.04 (63~99). The grip strength was 21.50 ± 5.03 (15~33) kg. The scores assessed at the same time were DASH 13.09 ± 7.43 (3~49) and PRWE 12.55 ± 11.41 (0~71).

**Fig. 3:**
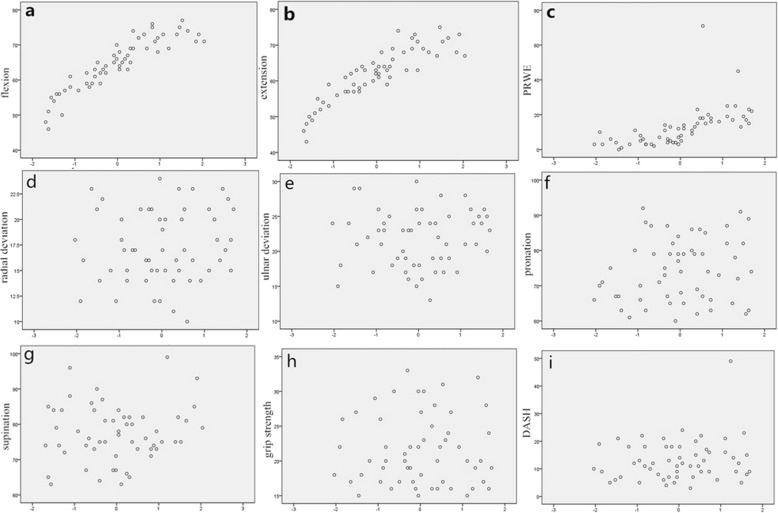
The scatterplots (**a**, **b**, **c**) showed the linear relationship between the ratios and flexion, extension, PRWE. No such correlation was found with radial deviation, ulnar deviation, pronation, supination, grip strength and DASH score (**d**, **e**, **f**, **g**, **h**, **i**)

After single factor linear regression analysis, the ratio was found having significant linear relationship with flexion degrees, extension degrees and PRWE scores. With the increase of the ratios, the flexion and extension range increased and the PRWE scores declined. Adjusted *R* squares were 0.82, 0.78, and 0.35 respectively for flexion degree, extension degree and PRWE score models and the corresponding *p*-values of the anova for the regression equation were all < 0.01. Regression coefficients were 43.39, 42.28, and − 43.09 respectively for the three models and the corresponding regression equations were as follows: y = 43.39x-14.46; y = 42.28x-15.15; y = 91.09–43.09x. Statistically significant results with all the *p*-values< 0.01 were also discovered when performing *t* tests for the regression coefficients. However, statistically significant linear relationship wasn’t found in radial deviation, ulnar deviation, pronation, supination, grip strength and DASH score (Fig. 3).

Complications occurred in 9 patients. Tendon damage in 4, traumatic arthritis in 2, and median nerve compression in 3 were observed after operation. There was no infection, nounion, malunion, implant loosening and breakage during the whole visits.

## Discussion

During daily work, a phenomenon was observed that if patients with DRF inherently had thick soft tissue but a thin radius in the wrist, the range of motion in flexion and extension after volar locked plating seemed better than others. We used the ratio of STCTBC to indicate the surrounding space between the skin surface and the bone surface at the watershed line in the distal radius and hypothesized that it has some relation with the range of motion of the wrist. Through this study, the hypothesis was completely demonstrated and a statistically significant linear relationship (*p* < 0.01) between the ratio and flexion-extension degree was discovered.

After injury, the soft tissue surrounding the wrist gets swollen. However, we measured the ratios based on the preoperative CT scans taken 5 to 7 days after the time of injury when the posttraumatic edema had completely subsided. So the ratio of STCTBC at the watershed line in the distal radius represents the patients’ inherent space between the bone surface and the skin in the distal radius without the impact of posttraumatic edema. A greater ratio means inherently larger space of the patients between the bone surface and the skin which may provide more effective buffer to avoid the damage to the wrist flexors and extensors. At the same time, larger space between the bone surface and the skin may further benefit the movement of the tendons and be more capable to avoid the impact between tendon and hardware. These may explain why the range of motion in flexion and extension augments with the increase of the ratios.

Although the correlation between the ratio and PRWE (r-squared value of 0.35) was not as close as flexion-extension degrees (r-squared value of 0.82, 0.78), the relationship was also statistically significant (*p* < 0.01). Perhaps PRWE related to many other factors and the ratio of STCTBC was just one among them.

Several factors have already been reported to predict the outcome of the volar plating system for the DRF. Age, AO classification, distal radial ulnar joint injury, ulnar styloid fracture and initial displacement are predictive of reduction loss and knowing that these factors are predictive can aid in early decision-making as to the method of treatment [10, 11]. Age, sex and size of dorsal cortex comminution can be used to predict the late dorsal tilt angulation of distal articular surface of radius at the end of the immobilization [12]. An increase in articular cavity depth and anteroposterior distance of the lunate fossa should be avoided when performing plate fixation to improve results following distal intra-articular radius fractures [13]. Flexor pollicis longus tendon rupture is a common complication leading to worse functional outcome after VLP for DRF. Selvan DR et al. [16] found that the risk of flexor pollicis longus tendon rupture decreased if the radial tilt was close to normal values after fracture reduction and the closer the distal end of the plate is to the joint, the risk of pollicis longus tendon ruptures increased. Kitay A et al. [17] also reported that plate position associated with attritional flexor tendon rupture following DRF with VLP. The rupture risk increased when plate prominence was greater than 2.0 mm volar to the critical line or plate position was within 3.0 mm of the volar rim.

Factors such as age, sex, distal radial ulnar joint involvement, fracture type and so on can be detected preoperatively while others like articular cavity depth, anteroposterior distance of the lunate fossa, position of plate and reduction quality can only be obtained intraoperatively or postoperatively. In our opinion, preoperative predictors make more sense for surgeons as they aid to make decision before operation. However, when using factors mentioned above such as age, sex, distal radial ulnar joint involvement, fracture type which can be detected preoperatively for prediction, it depends mainly on surgeons’ experience and there aren’t many specific standards. As a new method, the ratio is a kind of predictor which not only can be obtained preoperatively but also can be measured and calculated in the form of specific values. So it will help surgeons to foresee the wrist flexion-extension range of motion and even the final function more precisely without too much impact by surgeons’ subjective experience.

However, we failed to discover any relationship between the ratio and the other outcomes (pronation, supination, radial deviation, ulnar deviation, grip strength, DASH scores). We supposed that changes of the ratio dominantly mean alterations of the palmar and dorsal space in the wrist and have little relation with the radial and ulnar space as there is thicker soft tissue in the palmar and dorsal side than in the radial and ulnar side. In addition, when we perform the volar plate surgery, we put nothing in the radial or ulnar side and even we needn’t strip the soft tissue of both lateral sides. The hardware mainly occupies the palmar space and sometimes tips of the screws occupy the dorsal space. These may be the reasons why the ratio didn’t influence the pronation, supination, radial deviation and ulnar deviation degrees. Grip strength mainly depends on the muscle strength and many items of the DASH evaluation have much to do with the function of shoulder and elbow joint, so both of them can scarcely be influenced by the ratio.

However, this study is just exploratory and does have many limitations. Because of the obliquity and many other operational factors, it is impossible to obtain the planes 100% true, which affects the accuracy of the measurements. Lack of comparison to the opposite wrist caused that we didn’t know the motion range lost, and also, the difference in strength between the dominant and non-dominant hands. Although we could use the ratio to predict the outcomes of the VLP treatment for DRF, we didn’t know with what ratio patients should be surgically managed or just conservatively. Methodological improvements are deeply required to make the value as true as possible. Further subtle studies with comparison to the opposite wrist are absolutely needed to provide guideline for the surgeon regarding either the indication for surgery or the technique of volar locked plating.

## Conclusions

In conclusion, there is obvious linear relationship between the ratio of STCTBC at the watershed line and postoperative wrist flexion-extension degrees also PRWE scores when using volar locked plating for DRF. So the ratio can be used as a predictor aiding surgeons to predict the outcome.

## Data Availability

The datasets used and/or analysed during the current study are available from the corresponding author on reasonable request.

## Abbreviations

DASH: Disabilities of the arm, shoulder and hand
DRF: Distal radius fracture
PRWE: Patient-rated wrist evaluation
STCTBC: Soft tissue circumference to bone circumference
VLP: Volar locked plate
